# Lessons learned from different prognostic factors analysis in intermediate-risk neuroblastoma: ten years’ experience at a tertiary center

**DOI:** 10.1186/s43046-025-00335-6

**Published:** 2025-12-15

**Authors:** Mustafa Selim, Hanafy Hafez, Abeer M. Elsayed, Mohamed Shalaby, Abdalwahab R. Abdalwahab, Soad A. Eltokhy, Mohamed Fawzy

**Affiliations:** 1 Pediatric Oncology Department, National Cancer Institute (NCI), Cairo University, Giza, Egypt; 2 Cancer Pathology Department, National Cancer Institute (NCI), Cairo University, Giza, Egypt; 3 Surgical Oncology Department, National Cancer Institute (NCI), Cairo University, Giza, Egypt; 4 Clinical Pathology Department, National Cancer Institute (NCI), Cairo University, Giza, Egypt; 5 Children’s Cancer Hospital Egypt 57357, Cairo, Egypt

**Keywords:** IR-neuroblastoma, Induction therapy response, Surgical resection, Prognostic factors, Survival outcomes, Chemotherapy toxicity, Disease status

## Abstract

**Background:**

Intermediate-risk (IR) neuroblastoma represents a biologically and clinically diverse group of tumors. This study evaluates the feasibility of surgical excision and identifies prognostic factors that influence survival in IR-neuroblastoma patients, particularly those with suboptimal responses to induction chemotherapy.

**Methods:**

We conducted a retrospective analysis of 50 pediatric patients diagnosed with IR-neuroblastoma at a tertiary cancer center between 2007 and 2016. Treatment responses, surgical outcomes, and survival data were assessed. Prognostic variables were evaluated using univariable and multivariable models.

**Results:**

After four cycles of induction chemotherapy, 26% of patients showed an objective response, increasing to 62% by treatment completion. Surgical resection was performed in 70% of patients, with a higher proportion among non-responders. Initial response to induction chemotherapy was a significant independent predictor of surgical feasibility (*p* = 0.022) and final disease status (*p* = 0.026). Five-year overall survival (OS) was 84%, and event-free survival (EFS) was 72%. Surgical resection significantly improved end-of-treatment disease status in slow-responder patients but did not independently affect OS or EFS.

**Conclusion:**

Moderate-intensity chemotherapy with or without surgery provides acceptable survival outcomes in IR-neuroblastoma. An early favorable response to induction therapy may justify avoiding surgery, while surgical resection remains critical for slow-responder patients.

## Background

Neuroblastoma is the most common extracranial solid tumor in children, [1] accounting for approximately 15% of all childhood cancer deaths annually [2]. Its clinical behavior is highly variable, ranging from spontaneous regression to aggressive progression and chemoresistance [3]. Therefore, accurate risk stratification at diagnosis is essential for tailoring appropriate therapeutic strategies [4]. Intermediate-risk (IR) neuroblastoma typically involves localized or regional tumors with specific biological and clinical features that confer an intermediate probability of disease progression or relapse [5, 6]. Current guidelines recommend moderate-intensity chemotherapy combined with surgical tumor resection as the standard approach for IR-neuroblastoma, aiming to achieve excellent survival outcomes while minimizing long-term toxicities [5, 7].

Despite advances in molecular diagnostics and treatment protocols, variability in clinical outcomes persists even among patients classified under the same risk group [8]. Several studies have highlighted the importance of early response to induction therapy [5, 8], histopathological features [9, 10], chromosomal aberrations such as MYCN status and DNA ploidy [5ix, 13x], and the extent of surgical resection [11] as potential prognostic indicators. However, there is ongoing debate regarding the optimal timing and necessity of surgical intervention in infants who demonstrate a favorable initial response to chemotherapy [12].

This retrospective cohort study presents a 10-year experience at a tertiary cancer center in Egypt, focusing on the role of surgical resection in IR-neuroblastoma patients, particularly those with suboptimal responses to induction chemotherapy. Additionally, we evaluate various clinical and biological parameters as potential prognostic markers affecting survival outcomes. The findings aim to inform future treatment strategies by identifying key predictors of surgical feasibility and long-term prognosis.

## Methods

### Study design and population

This retrospective cohort study included all pediatric patients diagnosed with IR-neuroblastoma and treated at the Pediatric Oncology Department of a major tertiary care center, between January 2007 and December 2016. Patients were included if they were under 18 years of age and had confirmed neuroblastoma or ganglioneuroblastoma according to the International Neuroblastoma Pathology Classification (INPC). IR-neuroblastoma was defined based on the Children’s Oncology Group (COG) risk stratification criteria, [4] including stage 3 disease in patients < 18 months of age, infants (< 12 months) with stage 4 disease, stage 4 patients aged 12–18 months with favorable histology and hyperdiploidy, and infants with stage 4 s disease but unfavorable biological features such as diploidy or unfavorable histology. All patients underwent MYCN assessment through the use of fluorescence in situ hybridization in the tumor tissue samples at the time of diagnosis. Patients with low- or high-risk neuroblastoma or MYCN amplification or incomplete medical records were excluded.

### Procedure

#### Chemotherapy regimens

The first-line chemotherapy treatment involved 4 to 8 cycles of either regimen 1 (OJEC/OPEC) or regimen 2 (VP16-CARBO/CADO) for the patients. The first treatment plan alternated between OJEC therapy [vincristine 1.5 mg/m^2^ (max 2 mg), etoposide 200 mg/m^2^, carboplatin 500 mg/m^2^, cyclophosphamide 600 mg/m^2^ on day 1] and OPEC therapy [vincristine 1.5 mg/m^2^ (max 2 mg) with cyclophosphamide 600 mg/m^2^ on day 1 followed by cisplatin 90 mg/m^2^ on day 2 and etoposide 200 mg/m^2^ on day 3]. The VP16/CARBO component of Regimen 2 alternated between three days of etoposide 200 mg/m^2^ and carboplatin 450 mg/m^2^ on day 1 with the CADO component that included five days of cyclophosphamide.

Salvage chemotherapy with ICE (ifosfamide 1800 mg/m^2^ day 1–5, carboplatine 450 mg/m^2^ day 1, and etoposide 100 mg/m^2^ day 1–5) was administered in cases of progressive disease, non-response, or relapse, aiming for achieving response and/or improving surgical resectability.

#### Surgical intervention

After four cycles of induction chemotherapy, patients were evaluated for surgical resectability, and if not possible, they continued the same systemic chemotherapy till being operable. Surgery was attempted 2–3 weeks after completing the last cycle. Complete resection (100%), near-total resection (≥ 90%), or partial resection (< 90%) was performed depending on safety and feasibility. Surgery was omitted in cases of minimal residual disease or inoperable tumors.

#### Response evaluation

Tumor response was assessed post-induction, after local control, and at the end of treatment using the International Neuroblastoma Response Criteria (INRC), including complete response (CR): complete tumor disappearance, very good partial response (VGPR): decrease in primary tumor volume by 90–99%, partial response (PR): 50% to < 90% decrease in primary tumor volume compared to the measurement obtained at the time of diagnosis, stable disease (SD): no new lesions; more than 50% reduction of any measurable lesion with less than 50% reduction in any other, no response (NR): no new lesions; less than 20% reduction +/- less than 25% increase in any existing lesion, progressive disease (PD): any new lesion, increase of any measurable lesion by < 25%, or previous negative marrow became positive for tumor [6].

Objective response includes patients with CR or VGPR, while non-objective response includes patients with PR or worse. In the sub-analysis, patients with responses PR or less than PR after induction therapy were classified as slow-responder patients.

#### Endpoints

Primary endpoints included disease status at the end of treatment, overall survival (OS), and event-free survival (EFS). OS was calculated from the date of diagnosis to death or last follow-up. EFS was measured from diagnosis to disease progression, relapse, or death. Relapse was defined as new lesions or ≥ 25% increase in tumor size after initial treatment. Refractory disease was defined as non-response to any therapy, according to the INRC [6].

### Statistical analysis

Numerical variables were expressed as mean ± standard deviation or median with range, while categorical variables were summarized as frequencies and percentages. Comparisons were made using Student’s t-test for continuous variables and chi-square or Fisher’s exact test for categorical data. Survival outcomes were analyzed using the Kaplan-Meier method, and differences were assessed using the log-rank test. Multivariate Cox proportional hazards regression was used to identify independent prognostic factors. Hazard ratios (HRs) with 95% confidence intervals (CIs) were reported. All statistical tests were two-sided, with *p* < 0.05 considered statistically significant. IBM SPSS^®^ Statistics version 26 was used for data analysis.

## Results

### Patient characteristics

A total of 50 patients were included in the study, with an equal male-to-female ratio. Age at diagnosis ranged from 2 to 108 months, with a median of 11 months (± 17.6 SD). 60% of patients were younger than 12 months, and 84% had abdominal primary tumors. Histologically, 90% of cases were neuroblastoma, while 10% were ganglioneuroblastoma. Approximately two-thirds of patients were classified as stage 3 (66%), 26% as stage 4, and 6% as stage 4s. Histological classification (INPC) was unavailable in 8 patients (16%) due to insufficient tissue samples; however, their risk stratification remained unchanged based on other clinical parameters (Table 1).

**Table 1:** Characteristics of studied patients

Variable	Total (*n* = 50)	Slow response (*n* = 33)
Gender		
Male	25 (50)	19 (57.6)
Female	25 (50)	14 (42.4)
Age at time of diagnosis (years)		
*≤* 12 months	30 (60)	23 (69.7)
> 12 - ≤ 18 months	14 (28)	8 (24.2)
> 18 months	6 (12)	2 (6.1)
Site of primary tumor		
Abdominal	42 (84)	30 (90.9)
Mediastinal	6 (12)	3 (9.1)
Neck	1 (2)	---
Pelvic	1 (2)	---
Pathology		
Neuroblastoma	45 (90)	31 (93.9)
Ganglioneuroblastoma	5 (10)	2 (6.1)
INPC		
Favourable histology	27 (54)	18 (54.5)
Unfavourable histology	15 (30)	9 (27.3)
NA	8 (16)	6 (18.2)
Stage		
3	33 (66)	23 (69.7)
4	13 (26)	9 (27.3)
4s	3 (6)	1 (3)
NA	1 (2)	---
Stage 3		
*≤* 12 months	13 (39.4)	13 (56.5)
> 12 months - ≤ 18 months	14 (42.4)	8 (34.8)
> 18 months	6 (18.2)	2 (8.7)
First line systemic chemotherapy		
OJEC/OPEC	27 (54)	17 (51.6)
Vp16-CARBO/CADO	23 (46)	16 (48.4)
Disease status after induction treatment		
Objective response (CR/VGPR)	13 (26)	-----
Non-objective response (PR, less than PR, NR, PD)	37 (74)	28 PR − 5 Less than PR
Surgery		
Yes *	35 (70)	23 (69.7)
No	15 (30)	10 (30.3)
Second line systemic chemotherapy needed		
Yes	12 (24)	7 (21.2)
No	38 (76)	26 (78.8)
Cycles number in patients who didn’t need 2nd line treatment		
4 cycles	3 (8.1)	2 (7.7)
6 cycles	5 (13.5)	2 (7.7)
7–8 cycles	30 (78.4)	22 (84.6)
Disease status at the end of treatment		
Objective response (CR/VGPR)	31 (62)	19 (57.6)
Non-objective response (PR, less than PR, NR, PD)	19 (38)	14 (42.4)

Values are presented as number (%) unless otherwise indicated

*NA* Not assessed, *CR* Complete response, *INPC* International neuroblastoma pathology classification, *VGPR* Very good partial response, *PR* Partial response, *NR* No response, *PD* Progressive disease

*Unknown degree of surgical resection in 1 patient

### Treatment outcomes

Following induction chemotherapy (4 cycles), 26% of patients achieved an objective response (CR/VGPR), while 74% showed a non-objective response (PR or worse). By the end of treatment, 62% of patients achieved an objective response. Among the 33 patients (66%) classified as slow-responder patients post-induction, 57.6% achieved an objective response by treatment completion, while 42.4% remained with a non-objective response (Table 1).

The majority (93.9%) of slow-responder patients had neuroblastoma pathology, 90.9% had primary abdominal tumors, 69.7% were aged *≤* 12 months and had stage 3 disease, 84.6% received between seven and eight cycles of systemic chemotherapy, and 69.7% underwent surgical resection of the primary tumor (Table 1).

The type of post-induction treatment response (objective vs. non-objective) was not influenced by patient gender (male vs. female), age group (≤ 12 months, > 12 to ≤ 18 months, > 18 months), primary tumor location (abdomen, mediastinum, neck, or pelvis), tumor pathology (neuroblastoma vs. ganglioneuroblastoma), INPC classification (favorable vs. unfavorable), disease stage (3, 4, or 4 s), or chemotherapy regimen (1 vs. 2) (Table 2).

**Table 2:** Type of response after induction treatment in relation to different prognostic factors

Variable	Total (*n* = 50)	Non-objective (*n* = 37)	Objective (*n* = 13)	*P*-value
Gender				
Male	25 (50)	18 (72)	7 (28)	0.747
Female	25 (50)	19 (76)	6 (24)	
Age at time of diagnosis (years)				
*≤* 12 months	30 (60)	24 (80)	6 (20)	0.493
> 12 - ≤ 18 months	14 (28)	9 (64.3)	5 (35.7)	
> 18 months	6 (12)	4 (66.7)	2 (33.3)	
Site of primary tumor				
Abdominal	42 (84)	32 (76.2)	10 (23.8)	0.325
Mediastinal	6 (12)	4 (66.7)	2 (33.3)	
Neck	1 (2)	1 (100)	0	
Pelvic	1 (2)			
Pathology				
Neuroblastoma	45 (90)	34 (75.6)	11 (24.4)	0.452
Ganglioneuroblastoma	5 (10)	3 (60)	2 (40)	
INPC				
Favourable histology	27 (54)	21 (26.8)	6 (22.2)	0.732
Unfavourable histology	15 (30)	10 (66.7)	5 (33.3)	
NA	8 (16)	6 (75)	2 (25)	
Stage				
3	33 (66)	26 (78.8)	7 (21.2)	0.118
4	13 (26)	10 (76.9)	3 (23.1)	
4s	3 (6)	1 (33.3)	2 (66.7)	
NA	1 (2)			
First line systemic chemotherapy				
OJEC/OPEC	27 (54)	18 (66.7)	9 (33.3)	0.201
Vp16-CARBO/CADO	23 (46)	19 (82.6)	4 (17.4)	
Surgery (delayed) ^#^				
Yes	31 (62)	26 (70.3)	5 (38.4)	0.042
No	19 (38)	11 (29.7)	8 (61.6)	
Degree of surgical resection (*n* = 31)				
≥ 90% surgical resection	18 (58.1)	14 (53.85)	4 (80)	0.542
< 90% surgical resection	12 (38.7)	11 (42.3)	1 (20)	
NA *	1 (3.2)	1 (3.85)	0	
Second line systemic chemotherapy needed				
Yes	12 (24)	10 (27)	2 (15.4)	0.398
No	38 (76)	27 (73)	11 (84.6)	
Disease status at the end of treatment				
Objective response	31 (62)	20 (54)	11 (84.6)	0.051
Non-objective response	19 (38)	17 (46)	2 (15.4)	

Values are presented as number (%) unless otherwise indicated

*NA* Not assessed, *INPC* International neuroblastoma pathology classification

^#^Four patients underwent upfront surgical resection. ^*^Unknown degree of surgical resection in 1 patient. Objective response (complete response or very good partial response). Non-objective response (partial response or less than partial response or no response or progressive disease)

Regarding the impact of post-induction treatment response, patients who had an objective response to induction treatment were able to skip surgery more than those who did not (38.4% vs. 70.3%, respectively, *p* = 0.04). On the other hand, the degree of surgical resection (≥ 90% vs. <90% surgical resection), usage of 2nd line treatment ICE (yes vs. no), and disease status at treatment end (objective vs. non-objective) were not affected by post-induction treatment response and without statistically significant differences (*p* = 0.54, *p* = 0.39, and *p* = 0.051, respectively) (Table 2).

Surgical resection was performed in 70% of patients (*n* = 35), with 62.9% undergoing complete or near-complete resection (≥ 90%) and 34.3% having partial resection (< 90%). One case had an undocumented resection extent. Of the remaining 15 patients (30%) who did not undergo surgery, 26.7% (*n* = 4) had no/minimal residual disease (reached CR or VGPR responses by the end of the treatment), and 73.3% (*n* = 11) had inoperable tumors due to infiltration into adjacent structures or its encasement of critical blood vessels (ended up with the following responses: 3 CR, 3 PR, 1 NR and 4 PD). Among slow-responder patients (*n* = 33), 69.7% underwent surgical resection (*n* = 23)—13 had ≥ 90% resection, 9 had < 90%, and 1 had an undetermined extent of surgery. The remaining 10 patients (30.3%) did not undergo surgery due to an inoperable primary tumor (Table 1).

Patients with a primary tumor resection of ≥ 90% had a higher chance of achieving an objective response than those who underwent < 90% surgical resection (95.6% vs. 25%, respectively, *p* < 0.001), indicating that the degree of surgical resection had an impact on disease status at treatment end. Slow-responder patients had a statistically significant change in their disease status at treatment end depending on the extent of surgical resection and surgical treatment. Patients who underwent surgical removal of the primary tumor had a significantly higher objective response rate at the end of treatment (69.6%) compared to those who did not have surgery (30%; *p* = 0.035). Furthermore, the objective response rate reached 100% in patients who had ≥ 90% of the tumor excised, in contrast to just 33.3% in those with less than 90% resection (*p* = 0.001) (Table 3).

**Table 3:** Impact of surgery on disease and survival status at the end of treatment

All		Surgery		*P*-value	Degree of surgical resection			*P*-value
Variable	(*n* = 50)	Yes (*n* = 35)	No (*n* = 15)		≥ 90% (*n* = 22)	< 90% (*n* = 12)	NA * (*n* = 1)	
Disease status at the end of treatment for all patients								
Objective	31	24 (68.6)	7 (46.7)	0.144	21 (95.6)	3 (25)	0	< 0.001
Non-Objective	19	11 (31.4)	8 (53.3)		1 (4.4)	9 (75)	1 (100)	
Survival status at the end of treatment for all patients								
Alive	42	30 (85.7)	12 (80)	0.614	20 (90.9)	9 (75)	1 (100)	0.411
Dead	8	5 (14.3)	3 (20)		2 (9.1)	3 (25)	0	

Slow-responder patients		Surgery		P-value	Degree of surgical resection			P-value
Variable	(*n* = 33)	Yes (*n* = 23)	No (*n* = 10)		≥ 90% (*n* = 13)	< 90% (*n* = 9)	NA * (*n* = 1)	
Disease status at the end of treatment for patients with slow response								
Objective	19	16 (69.6)	3 (30)	0.035	13 (100)	3 (33.3)	0	0.001
Non-Objective	14	7 (30.4)	7 (70)		0	6 (66.6)	1 (100)	
Survival status at the end of treatment for all patients								
Alive	27	20 (87)	7 (70)	0.246	12 (92.3)	7 (77.8)	1 (100)	0.564
Dead	6	3 (13)	3 (30)		1 (7.7)	2 (22.2)	0	

Objective response (complete response or very good partial response). Non-objective response (partial response or less than partial response or no response or progressive disease)

*N* Number, *NA* Not assessed

^*^Unknown degree of surgical resection in 1 patient

Salvage chemotherapy (ICE) was required in 24% of patients (*n* = 12) because 5 patients had PD, 5 patients had NR, or 2 patients suffered relapse. Seven patients (58.3%) were under 12 months of age, and 83.3% had abdominal primaries and neuroblastoma pathology. Favorable vs. unfavorable histopathology, stage 3 vs. stage 4, and OJEC/OPEC vs. Vp16-CARBO/CADO regimen were equally distributed between the patients. Of these, 7 patients died (58.3%), and 5 survived, including one in CR, one in PR, two in NR, and one in PD.

### Survival outcomes

**Fig. 1:**
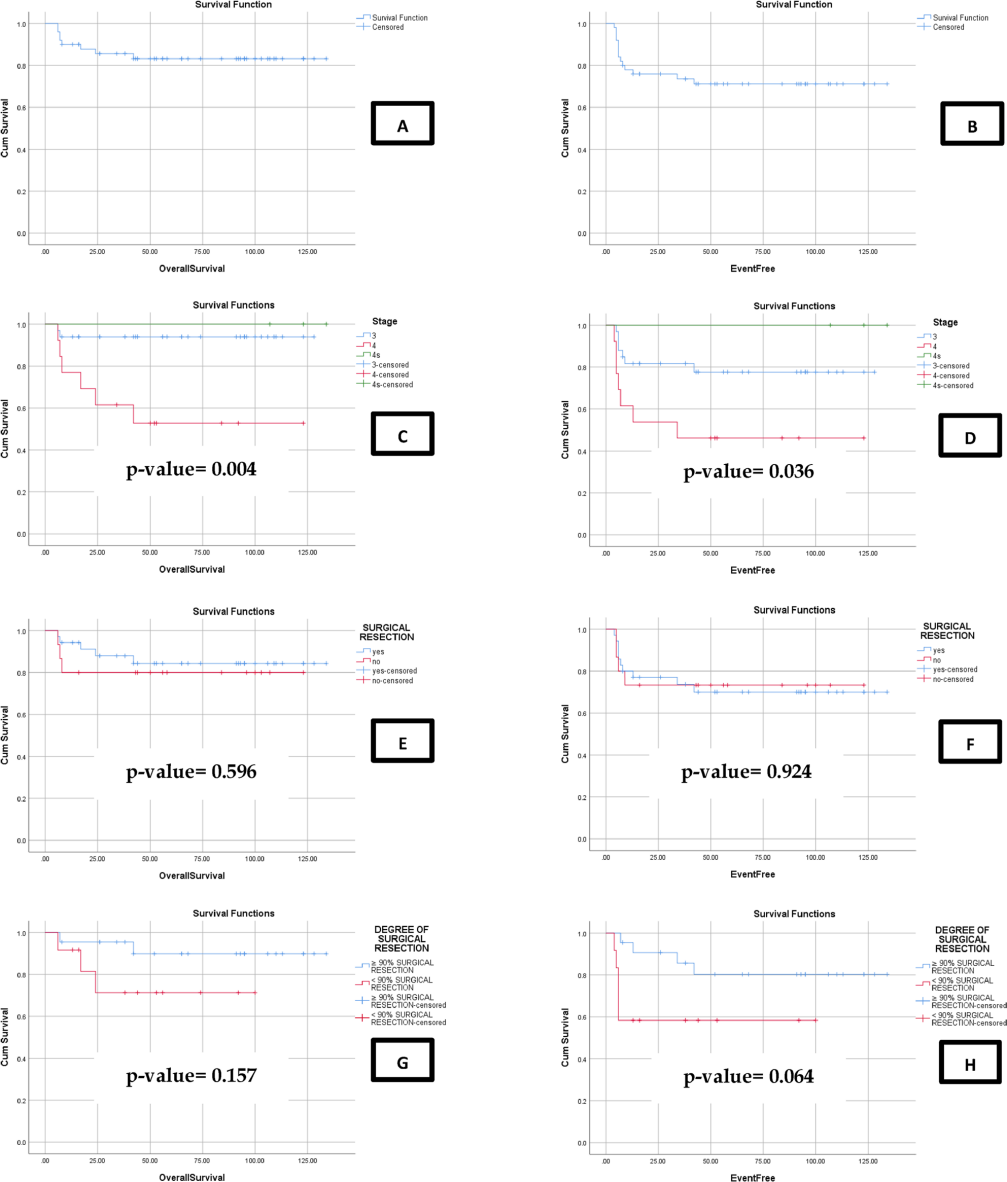
Kaplan-Meier survival curves for overall survival (OS) (**A**) and event-free survival (EFS) (**B**) among all studied patients with intermediate-risk neuroblastoma and OS and EFS according to different surgical stages (**C** & **D**); surgical resectability state(**E** & **F**); degree of surgical resection (**G** & **H**), respectively

With a median follow-up of 57 months (range: 6–134 months), the estimated 5-year OS was 84% ± 5.4%, and EFS was 72% ± 5.9%. Stage 4 patients had significantly lower OS (53.8% ± 14%) and EFS (46.2% ± 13%) compared to stages 3 and 4 s (Table 4; Fig. 1). However, this difference was not retained in multivariable analysis (Table 5).

**Table 4:** The estimated survival outcome for studied patients according to different prognostic factors

Variable	5- years OS				5- years EFS		
	*N*	OS (%)	95% CI	*p*-value	EFS (%)	95% CI	*p*-value
All	50	84	0.72–0.94	---	72	0.61–0.85	---
Age at time of diagnosis (years)							
**≤** 12 months	30	80	0.62–0.93	0.365	70	0.56–0.88	0.742
> 12 months	20	90	0.76–1.00		75	0.55–0.93	
Age at time of diagnosis (years)							
**≤** 18 months	44	81.8	0.68–0.92	0.28	72.7	0.61–0.87	0.632
> 18 months	6	100	---		66.7	0.29–1.00	
Gender							
Female	25	84	0.67–0.98	0.985	68	0.48–0.86	0.563
Male	25	84	0.68–0.98		76	0.63–0.95	
Stage							
3	33	93.9	0.85–1.00	0.004	78.8	0.68–0.95	0.036
4	13	53.8	0.25–0.80		46.2	0.19–0.73	
4s	3	100	---		100	---	
Stage 3							
**≤** 12 months	13	100	---	0.248	84.6	0.77–1.00	0.481
> 12 months	20	90	0.76–1.00		75	0.55–0.93	
Stage 3							
**≤** 18 months	27	92.6	0.82–1.00	0.501	81.5	0.71–0.98	0.307
> 18 months	6	100	---		66.7	0.29–1.00	
INPC							
Favourable	27	88.2	0.75–1.00	0.101	77.6	0.61–0.93	0.325
Unfavourable	15	65.2	0.40–0.90		59.3	0.34–0.84	
Disease status after induction treatment							
Objective response	13	84.6	0.65–1.00	0.863	84.6	0.65–1.04	0.212
Non-objective response	37	83.0	0.70–0.95		67.6	0.54–0.84	
Surgical resection							
Yes	35	85.7	0.79–0.92	0.596	71.4	0.66–0.77.9	0.924
No	15	80	0.70–0.89		73.3	0.63–0.83	
Degree of surgical resection (n, 34)*							
≥ 90% surgical resection	22	90.9	0.82–0.99	0.157	81.8	0.73–0.89	0.064
< 90% surgical resection	12	75	0.65–0.84		58.3	0.45–0.71	
Slow-responder patients	33	81.8	0.70–0.95	---	72.7	0.54–0.84	---
Surgical resection (n, 23)							
Yes	23	87	0.78–0.95	0.202	78.3	0.68–0.87	0.271
No	10	70	0.55–0.84		60	0.43–0.76	
Degree of surgical resection (n, 22)*							
≥ 90% surgical resection	13	92.3	0.79–1.00	0.313	84.6	0.19–1.00	0.564
< 90% surgical resection	9	77.8	0.50–1.00		77.8	0.35–0.92	

*CI* Confidence interval, *N* Number, *%* percentage, *OS* Overall Survival, *EFS* Event Free Survival, *CS* Cumulative Survival (%), *INPC* International neuroblastoma pathology classification

^*^Unknown degree of surgical resection in 1 patient. Objective response (complete response or very good partial response). Non-objective response (partial response or less than partial response or no response or progressive disease)

**Table 5:** Multivariate analysis of different prognostic factors for Intermediate-risk neuroblastoma

		Univariable	Multivariable	
Variable	Number	*p*-value	95% CI	*p*-value
Type of response after induction treatment				
Surgery (delayed) ^#^				
Yes	31 (62)	0.042	0.057 (0.005–0.659)	0.022
No	19 (38)			
Disease status at the end of treatment				
Objective response	31 (62)	0.051	7.453 (1.271–43.705)	0.026
Non-objective response	19 (38)			

Overall Survival				
Stage				
3	33	0.004	2.931 (0.935–9.188)	0.065
4	13			
4s	3			
Degree of surgical resection (n, 34)*				
≥ 90% surgical resection	22	0.157	1.286 (0.445–3.715)	0.642
< 90% surgical resection	12			

Event Free Survival				
Stage				
3	33	0.036	1.635 (0.662–4.300)	0.319
4	13			
4s	3			
Degree of surgical resection (n, 34)*				
≥ 90% surgical resection	22	0.064	1.247 (0.538–2.890)	0.607
< 90% surgical resection	12			

*CI* Confidence interval

^#^Four patients underwent upfront surgical resection, ^*^Unknown degree of surgical resection in 1 patient

Multivariate analysis revealed that initial response to induction therapy was a significant independent predictor of both surgical feasibility (*p* = 0.022) and disease status at treatment end (*p* = 0.026) (Table 5). Patients with an objective response to induction therapy were more likely to avoid surgery (38.4% vs. 70.3%, *p* = 0.04) (Table 2). Although the degree of surgical resection significantly affected disease status at treatment end (*p* < 0.001), it did not independently influence OS or EFS (Tables 4 and 5).

**Fig. 2:**
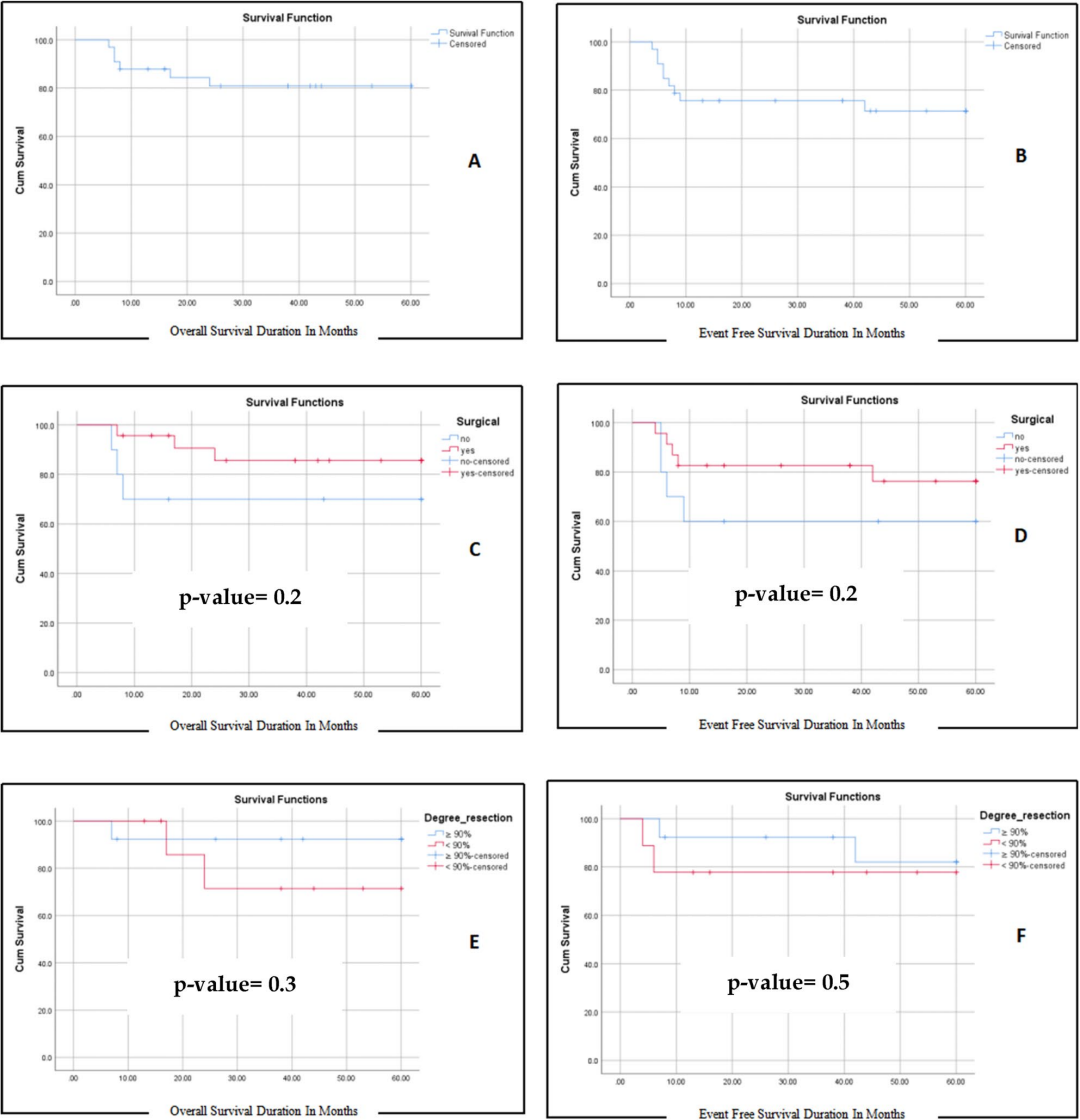
Kaplan-Meier survival curves for overall survival (OS) (**A**) and event-free survival (EFS) (**B**) among studied patients with the slow response to induction treatment and OS and EFS according to surgical resectability state (**C** & **D**) and degree of surgical resection (**E** & **F**), respectively

For slow-responder patients, the 5-year OS and EFS estimates were 81.8% ± 7.4% and 72% ± 8%. The extent of surgical resection did not change these numbers much. (Table 4; Fig. 2).

### Toxicity and mortality

Chemotherapy-related toxicity, according to the Common Terminology Criteria for Adverse Events (CTCAE) version 4.0, occurred in 22% of patients (*n* = 11), including septic shock (*n* = 6), ICU-requiring pneumonia (*n* = 2), hearing loss (*n* = 1), cardiac dysfunction (*n* = 1), and convulsions with altered consciousness (*n* = 1). No secondary malignancies were observed during follow-up. Eight deaths were recorded—six due to progressive disease, one with VGPR, and one with unknown disease status. All deaths were documented in the young age group (< 18 months, 6 patients with stage 4 and 2 patients with stage 3), and the majority (87.5%) after intensive second-line chemotherapy (ICE). Among slow-responder patients, mortality was higher in those who did not undergo surgery (30% vs. 13%) (Table 3).

## Discussion

This retrospective study provides insights into the management and prognostic factors influencing outcomes in pediatric intermediate-risk neuroblastoma over a 10-year period. Our findings confirm that moderate-intensity chemotherapy with or without surgery yields acceptable survival rates and manageable toxicity in this patient population [5, 7]. Importantly, the initial response to induction therapy emerged as the most significant independent predictor of a feasible resection and final disease status [5, 8, 9], suggesting that early response assessment should guide subsequent treatment decisions.

Although surgical resection was feasible in 70% of patients and significantly improved disease status at treatment end, particularly in slow-responder patients, it did not translate into improved OS or EFS in multivariable analysis. This finding aligns with previous reports indicating that the extent of resection does not always correlate with long-term survival, especially when effective systemic therapy is employed [5, 11, 12].

Our results also highlight the importance of individualized treatment planning, particularly in patients with poor initial responses to induction chemotherapy. These patients may benefit from more aggressive surgical interventions or intensified adjuvant therapies to improve disease control [5, 8, 12]. Conversely, patients demonstrating rapid and robust responses to induction therapy could potentially be managed with less invasive approaches, thereby reducing treatment-related morbidity [5, 7, 12].

While our study confirms the prognostic value of disease stage at presentation [5, 7], this significance was lost in multivariable analysis, underscoring the dynamic nature of neuroblastoma biology and the need for serial monitoring of treatment response [4, 5, 8]. Furthermore, despite known associations between histopathological features and outcome [5, 9, 10], subgroup analyses were limited by small sample sizes.

One limitation of this study is its retrospective design and relatively small sample size, which may limit the generalizability of findings [5, 7, 8]. Additionally, genetic profiling, including chromosome 11 segmental aberrations, was not uniformly available, potentially affecting risk stratification accuracy [4, 9, 10]. Finally, our limited resources, overcrowded rooms, and poor infection control can explain low survival outcomes among this young age group, which needs specific care and focus, especially those who are severely immunocompromised. Future prospective studies with larger cohorts and comprehensive molecular profiling are warranted to refine prognostic modeling and optimize treatment algorithms in IR-neuroblastoma.

## Conclusions

In conclusion, this study supports the use of moderate-intensity chemotherapy with selective surgical intervention in managing intermediate-risk neuroblastoma. The initial response to induction therapy serves as a crucial determinant of subsequent treatment strategy and outcome. Our observation that surgery may be safely be avoided in IR-neuroblastoma patients with favorable response to induction chemotherapy, based on eight patients in CR without surgery, (8/19, 41.1%), seems to be in line with findings in the literature.

## Data Availability

No datasets were generated or analysed during the current study.

## Abbreviations

CR: Complete Response EFS : Event-Free Survival
CTCAE: Common Terminology Criteria for Adverse Events
ICE: Ifosfamide, Carboplatin, Etoposide (chemotherapy regimen)
INPC: International Neuroblastoma Pathology Classification
IR-neuroblastoma: Intermediate-Risk Neuroblastoma
NA: Not Assessed
NR: No Response
OS: Overall Survival
OJEC: (Vincristine, Etoposide, Carboplatin, Cyclophosphamide)
OPEC: (Vincristine, Cyclophosphamide, Cisplatin, Etoposide)
PD: Progressive Disease
PR: Partial Response
VGPR: Very Good Partial Response
VP16-CARBO: (Etoposide, Carboplatin)
CADO: (Cyclophosphamide, Doxorubicin, Vincristine)
